# Fenofibrate improves endothelial function and plasma myeloperoxidase in patients with type 2 diabetes mellitus: an open-label interventional study

**DOI:** 10.1186/1758-5996-6-30

**Published:** 2014-03-04

**Authors:** Cristina Nita, Cornelia Bala, Mihai Porojan, Nicolae Hancu

**Affiliations:** 1 Department of Diabetes, Nutrition and Metabolic Diseases, “Iuliu Hatieganu” University of Medicine and Pharmacy Cluj-Napoca, 2-4 Clinicilor Street, 400006 Cluj-Napoca, Romania; 2 Department of Internal Medicine, “Iuliu Hatieganu” University of Medicine and Pharmacy Cluj-Napoca, 4-6 Clinicilor Street, 400006 Cluj-Napoca, Romania

**Keywords:** Fenofibrate, Myeloperoxidase, Endothelial dysfunction, sE-selectin, Type 2 diabetes

## Abstract

**Background:**

Fenofibrate offers a number of benefits on the cardiovascular system and it is plausible that its anti-inflammatory, anti-oxidant and anti-fibrotic effects and enhancement of cardiac metabolic performances may account for its direct cardioprotective effects.

In this study we aimed to investigate the effect of fenofibrate on endothelial function assesed by vascular studies and levels of soluble E-selectin (sE-selectin) as well as the effect on plasma myeloperoxidase (MPO) in patients with type 2 diabetes mellitus (T2DM) without previous use of lipid-lowering medication.

**Methods:**

27 patients (14 men and 13 women) with T2DM and good glycemic control (HbA1c: min 5.9%, max: 7.1%) treated with metformin monotherapy, without previous use of lipid-lowering medication were enrolled in this study. Vascular studies included measures of brachial artery diameter before and after release of a suprasystolic ischemia. FMD was calculated as the percent (%) change in arterial diameter following reactive hyperemia. Student’s paired t test and Wilcoxon Signed Ranks Test were used to compare values before and after fenofibrate therapy.

**Results:**

Fenofibrate therapy significantly increased post ischemia mean brachial artery diameter at 60 s (from 4.7 [4.4; 5.0] mm to 4.9 [4.6; 5.2] mm, p = 0.01) and at 90 s (from 4.7 [4.4; 5.0] mm to 4.9 [4.6; 5.1], p = 0.02). FMD response to hyperaemia at 60 s increased with 4.5 ± 13.7% (median value pre- treatment: 22.2%, median value post- treatment 25.0%, z = −2.9, p = 0.004). After 8 weeks of fenofibrate therapy, plasma MPO levels decreased to 49.5 [30.3; 71.5] ng/ml (% change from baseline = 4.6%, z = −2.2, p = 0.03) and mean plasma sE-selectin levels decreased to 67.1 [54.4; 79.8] ng/ml, (% change from baseline = 2.6%, p = 0.03).

**Conclusion:**

In patients with T2DM without previous treatment for dyslipidemia, short-term treatment with fenofibrate improved vascular endothelial function as demonstrated by increased post ischemia mean brachial artery diameter, increased FMD and decreased plasma sE-selectin and favorably affected plasma MPO levels. Therefore, fenofibrate may be considered a protective cardiovascular drug in this group of patients.

**Trial registration:**

(Australian New Zealand Clinical Trials Registry ANZCTR12612000734864)

## Background

Type 2 diabetes mellitus (T2DM) is associated with a high risk of cardiovascular (CV) events, irrespective of the presence of other traditional risk factors [1]. Current evidence suggests the central role of endothelium in all phases of the atherosclerotic process [2]. Most CV risk factors activate molecular pathways that result in increased expression of cytokines and cellular adhesion molecules involved in the adhesion and migration of monocytes into the subendothelial space [2,3], leading to the initiation, progression, and destabilization of the atherosclerotic lesion. E-selectin is a molecule of endothelial origin, which has been associated with carotid atherosclerosis and incident coronary heart disease [3,4]. Flow mediated dilation (FMD) evaluates endothelial dependent dilation in the brachial artery after occlusion, a measure of endothelial function in humans [5,6].

Myeloperoxidase (MPO) is a predominantly leukocyte-derived enzyme, involved in the initiation, destabilization of atherosclerotic plaque and genesis of acute coronary syndromes [7]. Plasma MPO levels have been positively associated with coronary artery disease (CAD) and risk of a subsequent cardiac event [8-11].

Therefore, drugs that have the potential to influence both vascular function and MPO have become highly interesting, especially if already used in the treatment of other CV risk factors in persons with diabetes. Fenofibrate offers a number of benefits on the CV system [12-14] and it is plausible that its anti-inflammatory, anti-oxidant and anti-fibrotic effects and enhancement of cardiac metabolic performances may account for its direct cardioprotective effects [15-17]. Previous studies showed that these effects could be mediated by decreased plasma high sensitivity C-reactive protein, fibrinogen, plasminogen activator inhibitor (PAI)-1 and decreased monocyte cytokine release [15], suppression of thromboxane A2 receptor, cytosolic calcium mobilization, and cyclooxygenase (COX)-1 activity [16] and decrease in inducible nitric oxide synthase, cyclooxygenase (COX)2 and matrix metallopeptidase 9 (MMP-9) [17].

The main purpose of this study was to investigate the role of fenofibrate on endothelial function, assessed by vascular studies and soluble E-selectin (sE-selectin), and on plasma MPO in patients with T2DM without previous use of lipid-lowering medication.

## Methods

### Study design and protocol

This was an interventional, open label study enrolling patients with T2DM treated with metformin alone, and without previous use of lipid-lowering medication. Participants were recruited from a T2DM population in an outpatient clinic from Romania. According to treatment protocols in place in Romania, each patient with diabetes treated with non-insulinic drugs must attend a specialist visit twice a year.

Participants were enrolled between August 1st and September 15, 2012 and followed for 8 weeks.

Each participant attended 3 study visits: screening, visits 1 and 2 (8 weeks apart). Starting from visit 1 participants fulfilling inclusion criteria and without any exclusion criteria, received 160 mg fenofibrate/day for 8 weeks. The pills were counted at the end of study; compliance was considered satisfactory if >90% of them were taken.

Before and at the end of the study, data were collected on medical history (visit 1), anthropometric variables, blood pressure (BP), FMD of the brachial artery, as well as adverse events (visit 2). Blood samples were collected at screening, visit 1 and 2 in fasting conditions as described below. At visit 1 each participant received instructions to maintain his/her usual nutritional habits and not to modify any drug treatment throughout the study.

The research was conducted in accordance with the guidelines in The Declaration of Helsinki and Good Clinical Practice. Study protocol was approved by the Iuliu Hatieganu University of Medicine and Pharmacy Ethics Committee. Written informed consent was obtained from each participant prior to any study procedures.

### Study objectives were to assess

1. The variation of brachial artery diameters and of FMD response to hyperaemia, after 8 weeks of treatment with fenofibrate compared to pre-treatment values

2. The variation of plasma MPO and soluble sE-selectin after 8 weeks of treatment with fenofibrate as compared with pre-treatment values.

### Study population

We enrolled men and women ≥18 years of age, with T2DM defined according to World Health Organization criteria (WHO) [18], who were on stable doses of metformin for at least 3 months, with glycated haemoglobin (HbA1c) <7.5% and without previous treatment with any lipid-lowering drugs.

Exclusion criteria were: known type 1 and other specific types of diabetes (e.g. genetic defects of the β-cell, genetic defects in insulin action, diseases of the exocrine pancreas, endocrinopathies, drug- or chemical-induced diabetes, infections, uncommon forms of immune-mediated diabetes, other genetic syndromes associated with diabetes) according to WHO classification of diabetes mellitus [18], treatment with other hypoglycaemic drugs during the 3 months preceding the screening visit, any acute cardiovascular event within last 3 months, genetic conditions affecting lipids metabolism (e.g. familial hypercholesterolemia, lipoprotein lipase deficiency), uncontrolled endocrine or metabolic diseases, chronic kidney disease (glomerular filtration rate [eGFR] <50 ml/min/1.73 m^2^, contraindicating treatment with fenofibrate), serum triglyceride level ≥400 mg/dl, hepatic enzymes >3 upper normal limits (UNL), history of alcohol abuse, changes in antihypertensive therapy during the last 3 months.

### Data collection and laboratory tests

A complete medical history, including diabetes duration and treatment, was obtained for each participant; height, weight and waist circumference were determined by a standardized protocol. Waist circumference was measured at half of the distance between the lower border of the last rib and the upper border of the iliac crest at the end of a normal expiration, using a non-stretchable tape measure. Body mass index (BMI) was calculated as weight (kg)/[height (m)]^2^. Blood pressure was measured using a calibrated standard mercury sphygmomanometer. All readings were taken after a 5-min rest, in the sitting position.

Fasting blood samples were collected in the morning after an 8 h overnight fasting period. Fasting plasma glucose, HbA1c, total cholesterol, high-density lipoprotein (HDL) cholesterol, triglycerides, creatinine, hepatic enzymes were determined by automated chemistry analyzer Konelab 30, Thermo Fisher Scientific Inc, Finland. Low-density lipoprotein (LDL) cholesterol was calculated using Friedewald formula [19]. eGFR was estimated using online available Modification of Diet in Renal Disease (MDRD) calculator (http://www.nephron.com/MDRD_GFR.cgi). sE-selectin and plasma MPO were measured in plasma (EDTA) using commercially available enzyme-linked immunosorbent assay kits for quantitative detection (DRG Diagnostics GmbH, Germany and DRG International, Inc, USA) on an automated ELISA reader (Tecan).

Adverse events were recorded throughout of study. Safety parameters included serum creatinine, eGFR, and hepatic enzymes.

### Vascular studies

Imaging studies of the right brachial artery were performed in fasting condition before and after 8 weeks of fenofibrate therapy with a high-resolution, ultrasound imaging system (Aloka ProSound Alpha 10 Premier, Aloca Co., Ltd.) using M-mode, and electrocardiogram-triggered ultrasound images obtained with a 10 MHz linear-array transducer. All vascular studies were performed according to a protocol previously described by Celermajer and co-workers [5], by measuring the arterial response to reactive hyperemia. Studies were performed at 20–24°C in a dark, quiet room. The study participant rested for at least 10 min before the first scan and remained in a recumbent position throughout the investigation. Scanning of the brachial artery was performed 3–10 cm above the elbow, at baseline (before ischemia) and at 45, 60, 90, and 120 s after release of a suprasystolic ischemia of the forearm with a pneumatic cuff, inflated at 250 mmHg for 4 min. Images were taken at the end of the diastole and digitalized. Four cardiac cycles were analyzed at the end of the diastole, and arterial diameter was automatically measured using special software (E-tracking) and then averaged. FMD was calculated as the percent (%) change in arterial diameter following reactive hyperemia when compared to the baseline diameter.

### Statistical analysis

Statistical analysis was performed using SPSS-PC 15.0 (SPSS Inc., Chicago, IL, USA). Data were considered to be normally distributed if the ratio of skewness to its standard deviation did not exceed the value of 2. Data were expressed as a mean [95% Confidence Interval] for variables with normal distribution, median [1st quartile; 3rd quartile] for variables with abnormal distribution or frequency for dichotomial variables. Student’s paired t test (for variables with normal distribution) and Wilcoxon Signed Ranks Test (for variables with deviations from normal distribution) were used to compare values before and after fenofibrate therapy. Correlations were assessed with Pearson’s and Spearman’s correlation coefficient analysis. The level of significance was set at 0.05, and all tests were performed two- sided.

## Results

### Baseline characteristics

Twenty seven patients (14 men and 13 women) fulfilling the inclusion criteria and without any exclusion criteria were enrolled in this study. Two participants (one male and one female) did not complete the study: one was excluded from the study due to poor compliance with fenofibrate treatment and one dropped out soon after fenofibrate treatment initiation for personal reasons and refused to present for reevaluation. Mean duration of diabetes was 3.8 years. All participants had good glycemic control (HbA1c range: 5.9%-7.1%). Doses of metformin ranged from 850 to 3000 mg/day. 76.0% of study participants had a history of hypertension and 20.0% had cardiovascular diseases. Characteristics of study participants are presented in Table 1.

**Table 1:** Characteristics of the 25 patients at baseline and at follow up (8 weeks of treatment)

**Variables**	**Visit 1 (Pre-treatment)**	**Visit 2 (Post treatment)**	**p**
Age (years)	59.2 [55.2; 63.2]	-	
Male gender (%)	13.0 (52.0%)	-	
Current smoker	4.0 (16.0%)	-	
Hypertension	19.0 (76.0%)	-	
Cardiovascular disease	5.0 (20.0%)	-	
Ischemic heart disease	4.0 (16.0%)	-	
Ischemic stroke	1.0 (4%)	-	
Medication			
ACEI	10.0 (40.0%)	-	
β-Adrenergic receptor blocker	8.0 (32.0%)	-	
Diuretics	8.0 (32.0%)	-	
Calcium channel blocker	7.0 (28.0%)	-	
Angiotensin receptor blocker	4.0 (16.0%)	-	
Aspirin	7.0 (28.0%)	-	
SBP (mmHg)	149.8 [143.5; 156.1]	142.7 [137.6; 147.8]	0.04
DBP (mmHg)	91.5 [87.4; 95.5]	87.5 [82.8; 92.2]	0.02
BMI (kg/m^2^)	28.7 [26.6; 34.2]^*a*^	28.7 [26.7; 34.0]^*a*^	0.79
Waist (cm)	108.5 [104.0; 113.0]	110.4 [105.5; 115.3]	0.06
FPG (mg/dl)	136.9 [129.0; 144.8]	137.6 [129.7; 145.5]	0.82
HbA1c (%)	6.5 [6.3; 6.8]	6.5 [6.3; 6.7]	0.53
Lipids			
Total cholesterol (mg/dl)	182.0 [166.0; 220.0]^*a*^	168.0 [151.0; 192.5]^*a*^	0.01
HDL cholesterol (mg/dl)	45.0 [40.1; 50.0]	44.1 [37.7; 50.5]	0.88
LDL-cholesterol (mg/dl)	104.0 [93.0; 139.3]^*a*^	102.0 [89.1; 125.6]^*a*^	0.14
Triglycerides (mg/dl)	168.6 [140.2; 197.0]	114.1 [95.4; 132.8]	<0.001
eGFR (ml/min/1.73 m^2^)	107.7 [93.4; 122.0]	96.8 [82.6; 111.1]	0.007
TGO (IU/L)	20.0 [15.5; 25.0]^*a*^	22.0 [18.0; 26.0]^*a*^	0.10
TGP (IU/L)	22.0 [17.0; 27.0]^*a*^	24.0 [20.0; 28.0]^*a*^	0.48

Data in table are presented as mean [95%CI] for continuous variables, median [1st quartile; 3rd quartile] for variables with abnormal distribution and number (%) for categorical variables; ^a^- abnormal distribution; BMI - body mass index; HbA1c – glycosilated hemoglobin; SBP –systolic blood pressure; DBP – diastolic blood pressure; FPG – fasting plasma glucose; eGFR- estimated glomerular filtration rate.

Compared with baseline, at visit 3 participants presented significantly lower levels of total cholesterol (−24.3 mg/dl, p = 0.002), triglycerides levels (−54.9 mg/dl, p < 0.001), systolic and diastolic blood pressure (−6.8 mmHg, p = 0.04 and −3.8 mmHg, p = 0.02, respectively). No statistically significant effect was observed on HDL-cholesterol and on LDL-cholesterol (p > 0.05). In addition, no change was observed in fasting plasma glucose, HbA1c, BMI and waist after fenofibrate treatment (Table 1, p >0.05).

### FMD

**Figure 1:**
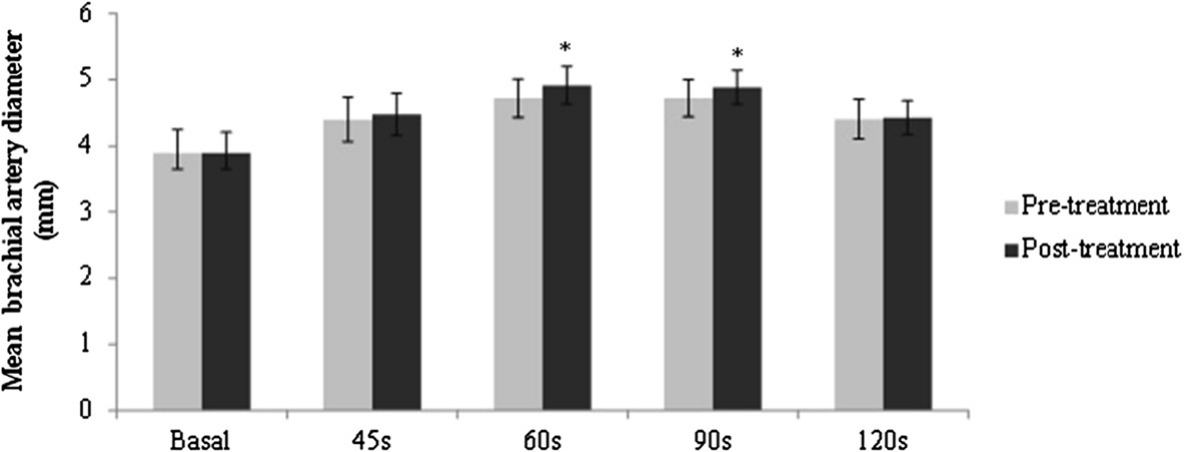
**Effect of fenofibrate on brachial artery diameter in basal conditions and during hyperemia at 45, 60, 90 and 120 seconds.** *Statistically significant difference in mean brachial artery diameter pre- and post-treatment. Error bars represent 95% confidence intervals Basal, before hyperemia; 45 s, hyperemia 45 s; 60 s, hyperemia 60 s; 90 s, hyperemia 90 s; 120 s, hyperemia 120 s.

The variation of brachial artery diameters before and after treatment is shown in Figure 1. Mean basal brachial artery diameter was similar at each ultrasound assessment, with no significant difference between pre-treatment and post-treatment diameter (p = 0.34). Post ischemia mean brachial artery diameter at 60 s and 90 s increased significantly after fenofibrate therapy: from 4.7 [4.4; 5.0] mm to 4.9 [4.6; 5.2] mm at 60 s, p = 0.01 and from 4.7 [4.4; 5.0] mm to 4.9 [4.6; 5.1] at 90 s, p = 0.02). No significant differences between pre-treatment and post-treatment diameter were observed at 45 s and 120 s (p = 0.18 and 0.67, respectively).

**Figure 2:**
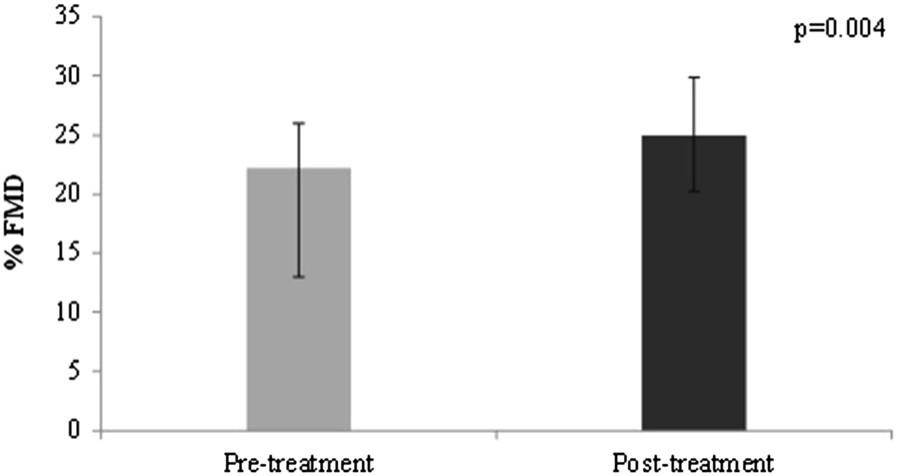
**Effect of fenofibrate on percent flow-mediated dilation.** Bars represent median values; error bars represent quartile 1 and quartile 3% FMD, percent flow-mediated dilation.

Additionally, compared with pre-treatment values, FMD response to hyperaemia at 60 s increased with 4.5 ± 13.7% (median value pre- treatment: 22.2%, median value post- treatment 25.0%, z = −2.9, p = 0.004) (Figure 2).

No correlations were observed between changes in FMD and changes in total cholesterol (Spearman correlation coefficient ρ = −0.1, p = 0.50), triglycerides (ρ = −0.3, p = 0.07) or changes in systolic BP (ρ = −0.2, p = 0.37) or diastolic BP (ρ = −0.08, p = 0.72).

### MPO and sE-selectin

**Figure 3:**
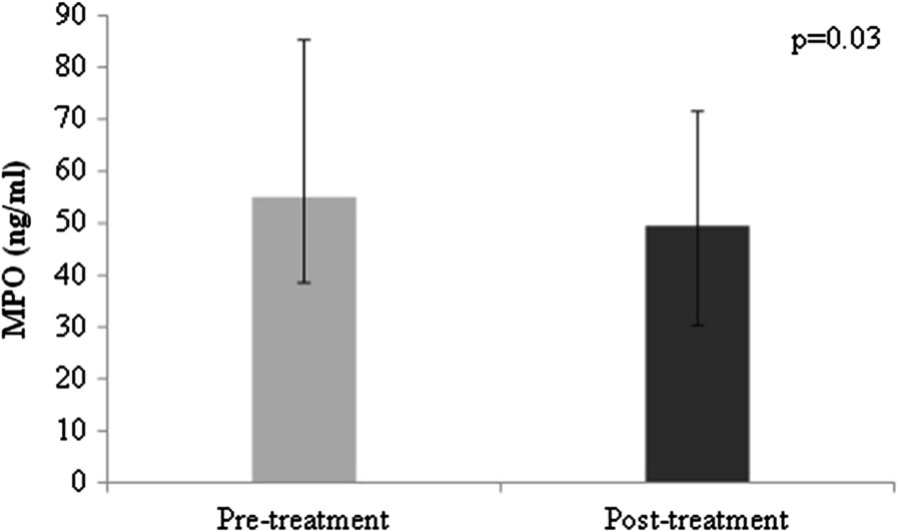
**Myeloperoxidase levels (ng/ml) before and after treatment with fenofibrate.** Bars represent median values; error bars represent quartile 1 and quartile 3 MPO, myeloperoxidase.

**Figure 4:**
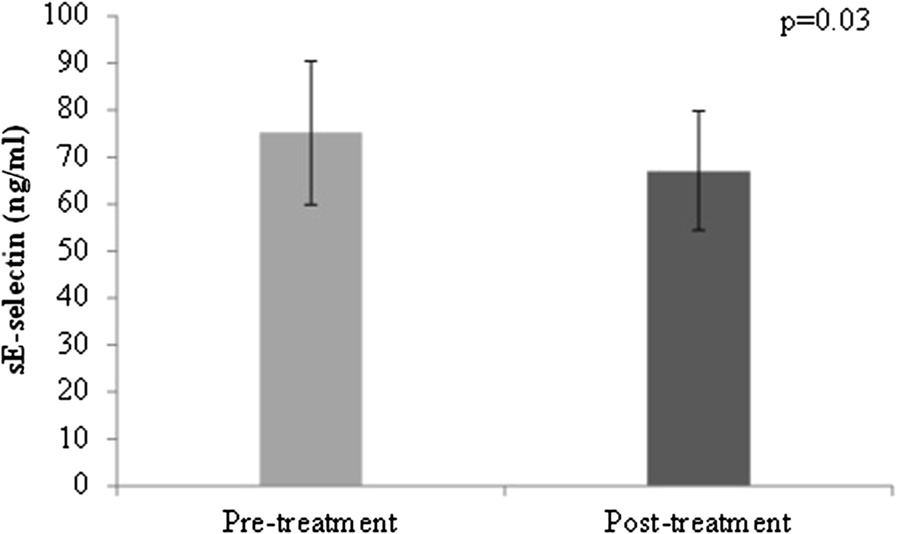
**sE-selectin levels (ng/ml) before and after treatment with fenofibrate.** Error bars represent 95% confidence intervals.

At baseline, MPO values ranged from 16.5 to 236.5 ng/ml, with a median level of 55.0 [38.5; 85.3] ng/ml and values of sE- selectin ranged from 15.0 to 142.0 ng/ml, with a mean level of 75.2 [59.9; 90.5] ng/ml. After 8 weeks of fenofibrate therapy, plasma MPO levels decreased to 49.5 [30.3; 71.5] ng/ml (% change from baseline = 4.6%, z = −2.2, p = 0.03) (Figure 3) and mean plasma sE-selectin levels decreased to 67.1 [54.4; 79.8] ng/ml (% change from baseline = 2.6%, p = 0.03) (Figure 4).

### MPO and endothelial dysfunction

No correlation was observed between % change in MPO levels and % change in FMD (Spearman correlation coefficient ρ = 0.3, p = 0.18) or between % change in MPO levels and % change in E selectin (Spearman correlation coefficient ρ = −0.2, p = 0.29).

### Safety parameters

No serious adverse effects were observed throughout the study, and none of the patients withdrew due to adverse effects. A statistically significant decrease of eGFR occurred between baseline and final visit (107.7 [93.4; 122.0] vs. 96.8 [82.6; 111.1] ml/min/1.73 m^2^, p = 0.007) (Table 1).

## Discussions

In this study, we showed that fenofibrate improved endothelial dysfunction in T2DM patients without previous use of lipid-lowering medication and that this improvement was not related to changes of the lipid profile or blood pressure. We also showed that fenofibrate reduced plasma MPO concentrations.

Our results demonstrated that short term treatment with fenofibrate significantly decreased total cholesterol and triglycerides concentrations. Additionally, we showed that it significantly improved post ischemia brachial artery diameter and FMD, an effect which was independent of the reduction in plasma lipids and blood pressure, decreased plasma MPO and sE-selectin levels and BP values.

### Effect of fenofibrate on lipid profile

Regarding the effect of fenofibrate on plasma triglycerides and total cholesterol, our results are consistent with those of large clinical trials, showing that fenofibrate therapy in patients with dyslipidemia is associated with significant reductions of triglycerides, total and LDL-cholesterol levels, and increases HDL-cholesterol levels [12,20-25]. We did not observe any improvement in LDL and HDL- cholesterol levels. This may be explained by the normal lipid levels observed in most of the participants enrolled in our study.

### Effect of fenofibrate on blood pressure

The results of our study showed a small but significant reduction in both systolic and diastolic BP. In several animal models it was shown that fenofibrate may have a favorable effect on this parameter. Shatara et al. demonstrated that fenofibrate reduced blood pressure in stroke-prone spontaneously hypertensive rats and Dahl salt-sensitive rats [26]. Also, fenofibrate prevented the development of hypertension, myocardial inflammation and fibrosis in Ang-II-infused rats [27]. However, no data in humans have been published.

### Effect of fenofibrate on vasomotor function (FMD and sE-selectin)

The results of our study revealed that fenofibrate therapy significantly improved post ischemia brachial artery diameter and %FMD. This finding is consistent with other studies that have shown significantly improved FMD response to hyperemia in patients with metabolic syndrome or hypertriglyceridemia following 6–8 weeks of fenofibrate administration [28-30]. A similar effect, partially related to enhanced reduction in LDL-cholesterol and apoB-100 concentrations, has been shown in T2DM patients treated with statins [31]. It was also observed that short-term treatment with fenofibrate improves vascular endothelial function in healthy normolipidemic middle-aged and older adults by reducing oxidative stress and induces an increase in endothelial nitric oxide synthase [13].

A favorable impact of 8-week treatment with fenofibrate on FMD in T2DM patients with typical or mixed dyslipidemia has recently been reported [32], and this effect was independent of reduction in lipid parameters. These findings are concordant with our results, suggesting that in patients with T2DM fenofibrate could have positive effects on endothelial dysfunction beyond its antihyperlipidemic action. It cannot be excluded that more subtle changes such as LDL cholesterol particle size could be, at least in part, responsible for improvement in endothelial function with fenofibrate.

We cannot completely exclude that changes in systolic and diastolic BP had an impact on FMD, despite lacking correlations between the three parameters. The interpretation of this possible relationship is limited by the study design which was not placebo-controlled.

Regarding the improvement in sE-selectin levels seen in our study, the results are discordant with those derived from the Fenofibrate Intervention and Event Lowering in Diabetes (FIELD) trial and other studies showing that both long-term and short-term fenofibrate treatment have no effect on this marker of arterial damage [33,34].

Observational studies have suggested that there is a strategic accumulation of MPO at the interface between endothelium and media. This observation led to the hypothesis that MPO could affect vascular tone by depleting endothelium derived NO [35,36]. Subendothelial accumulated MPO can act locally as an NO oxidase, inhibiting NO function and causing endothelial dysfunction [37]. The recently published work of Rudolph et al. demonstrated that MPO elicits profound effects on vascular tone of conductance and resistance vessels both in humans, and in animal models [38]. Their results showed a significant inverse correlation between FMD and MPO plasma levels prior to and following nicotine application (r = −20.33, P = 0.03; r = −20.30, P = 0.04) [38]. Another research conducted by Vita et al. suggested that there was a strong, independent relation between serum MPO level and endothelial dysfunction, as reflected by brachial artery flow–mediated dilation [39]. By contrast, we observed that there was no correlation between % changes in MPO levels and % change in FMD or % changes in E selectin, suggesting that changes in FMD and E selectin were independent from changes in MPO levels and could be directly attributed to fenofibrate therapy.

A possible explanation for the favorable effects of fenofibrate on endothelial function in T2DM, independent of its antihiperlipidemic action, is that this drug has direct anti-inflammatory and anti-oxidant effects on vascular wall, a hypothesis that was partially confirmed in healthy normolipidemic subjects [13] but not yet tested in patients with diabetes.

### Effect of fenofibrate on plasma myeloperoxidase

To our knowledge, no prior human study has examined the effect of fenofibrate on plasma MPO concentration. Given that MPO adversely influences LDL atherogenic properties and HDL functionality, it may be hypothesized that drugs reducing this enzyme level could have certain benefits in reducing the risk for atherosclerotic events.

Experimental and clinical studies provide support for a potential impact of statin therapy on tissue and plasma MPO levels both in animal models and in humans [40-43]. In clinical studies, statins reduced circulating levels of MPO in patients with congestive heart failure [40], acute coronary syndromes [41] and patients with diabetes on dialysis [42]. The study conducted by Ndrepepa et al. demonstrated an association between therapy with beta-blockers on admission and reduced circulating levels of plasma MPO which was independent of cardiovascular risk factors, clinical characteristics and concomitant therapy in a consecutive series of patients with symptomatic CAD [43].

If our results regarding the beneficial effect of fenofibrate on plasma MPO and on endothelial function will be confirmed in larger populations of patients with T2DM, this drug could become a valuable option for reducing atherosclerotic process in this group of high cardiovascular risk patients as part of an early and comprehensive preventive strategy.

### Safety issues

Our results regarding the decline in renal function are concordant with those in the FIELD and Action to Control Cardiovascular Risk in Diabetes (ACCORD) studies showing that fenofibrate caused an acute, sustained plasma creatinine increase [25,44]. In a recent FIELD sub study, Davies et al. assessed fenofibrate’s renal effects and found no evidence that the initial increase in plasma creatinine levels represented true renal injury, a finding that has important implications for clinical care [45]. Moreover, it seems that despite initial and reversible increase in plasma creatinine levels, fenofibrate may delay albuminuria and GFR impairment in T2DM patients [46].

### Limitations of the study

The main limitations of the present study are the relatively small number of patients included and the short duration of the fenofibrate treatment.

## Conclusions

In patients with T2DM without previous treatment for dyslipidemia, short-term treatment with fenofibrate improved vascular endothelial function as demonstrated by increased post ischemia mean brachial artery diameter, increased FMD and decreased plasma sE-selectin and favorably affected plasma MPO levels. Therefore, fenofibrate may be considered a protective cardiovascular drug in this group of patients.

## Abbreviations

BMI: Body mass index; BP: Blood pressure; CAD: Coronary artery disease; CV: Cardiovascular; eGFR: Estimated glomerular filtration rate; FMD: Flow mediated dilation; HbA1c: glycated hemoglobin; HDL: High density lipoprotein; LDL: Low density lipoprotein; MPO: Myeloperoxidase; NO: Nitric oxide; sEselectin: Soluble eselectin; T2DM: Type 2 diabetes mellitus; WHO: World Health Organization.

## Competing interests

The authors declare that they have no competing interests.

## Authors’ contributions

CN and CB were study coordinators, participated in the design of the study, were responsible for individual selection and characterization, carried out the statistical analysis and drafted the manuscript. MP carried out vascular studies and participated to manuscript preparation. NH participated in the design of the study and to manuscript preparation. All authors read and approved the final manuscript.
